# In the article “Prevalence and exposure variables of latent infection by mycobacterium tuberculosis in healthcare workers”

**DOI:** 10.1590/0034-7167.20257804e05

**Published:** 2025-09-01

Where it read:

(...) however, in the study by Bukhary et al.^(14)^, an association with LTBI was found in the IGRA test, with females being more prevalent than males, 94.2% and 86.8%, respectively (p = 0.01). Conversely, Coppeta et al.^(24)^ showed in their analysis that males were related to LTBI with twice the prevalence compared to females (p = 0.01).

Read:

(...) however, in the study by Bukhary et al.^(14)^, an association with LTBI was found in the IGRA test, with males being more prevalent than females, 13,2% and 5,8%, respectively (p = 0.01). In the same vein, Coppeta et al.^(24)^ showed in their analysis that males were related to LTBI with twice the prevalence compared to females (p = 0.01).

