# Supplementary material for: In the article “Prevalence and exposure variables of latent infection by mycobacterium tuberculosis in healthcare workers”
Source: Rev Bras Enferm. 2025 Sep 1;78(4):e2025n4e05. doi: 10.1590/0034-7167.20257804e05 (PMC12407880; doi:10.1590/0034-7167.20257804e05)
Supplement: Related texts

# Correction: No artigo “Prevalência e variáveis de exposição da Infecção Latente pelo Mycobacterium tuberculosis em Trabalhadores de Saúde”

**DOI:** 10.1590/0034-7167.20257804e05pt

Onde se lia:

(...) já no estudo de Bukhary et al^(14)^, foi achada associação à ILTB no teste IGRA, sendo o sexo feminino mais prevalente que o sexo masculino 94,2% e 86,8% respectivamente (p=0,01); ao contrário, Coppeta et al^(24)^, mostrou em sua análise que o sexo masculino está relacionado à ILTB com o dobro da prevalência se comparado ao sexo feminino ( p=0,01).

Lê-se:

(...) já no estudo de Bukhary et al^(14)^, foi achada associação à ILTB no teste IGRA, sendo o sexo masculino mais prevalente que o sexo feminino 13,2% e 5,8% respectivamente (p=0,01); No mesmo sentido, Coppeta et al^(24)^, mostrou em sua análise que o sexo masculino está relacionado à ILTB com o dobro da prevalência se comparado ao sexo feminino (p=0,01).
